# Quality Indicators of Pharmaceutical Care for Integrative Healthcare: A Scoping Review of Indicators Developed Using the Delphi Technique

**DOI:** 10.1155/2020/9131850

**Published:** 2020-03-19

**Authors:** Ramzi Shawahna

**Affiliations:** ^1^Department of Physiology, Pharmacology, and Toxicology, Faculty of Medicine and Health Sciences, An-Najah National University, Nablus, State of Palestine; ^2^An-Najah Biosciences Unit, Centre for Poisons Control, Chemical and Biological Analyses, An-Najah National University, Nablus, State of Palestine

## Abstract

**Background:**

Recently, measuring and benchmarking provision of healthcare services has drawn a considerable attention. This scoping review was conducted to identify, describe, and summarize studies in which the Delphi technique was used to develop quality indicators of pharmaceutical care. The study also aimed to identify activities and services that could be used to capture the impact of pharmacist in integrative medicine.

**Methods:**

Databases were searched from inception to February 2020 using key terms that were combined using Boolean operators. Studies were included if they were relevant to development of quality indicators of pharmaceutical care with regard to medications or complementary and alternative medicine (CAM) modalities. Full text of the selected studies was imported into EndNote. Studies were screened and data were extracted into a standard extraction form.

**Results:**

Data were extracted from 31 studies. Of those, 24 (77.4%) were related to provision of pharmaceutical services relevant to medications and 7 (22.6%) were related to provision of care using CAM modalities. Most of the studies (67.7%) were published in 2010 and beyond. Almost half of the studies (48.4%) originated from the United States, United Kingdom, and Canada. A total of 42 different activities and services that could be used as quality indicators were identified from the studies included in this review. Activities and services were related to history taking, performing reconciliations, identifying and resolving therapy problems, providing collaborative care, designing care plans, optimal performance, and continuing education.

**Conclusions:**

Although there is an increasing interest in improving healthcare delivery, quality indicators of pharmaceutical services and those relevant to CAM provision in healthcare facilities adopting the integrated healthcare paradigm are still limited. Future studies are needed to develop validated quality indicators that could be successfully used in measuring and benchmarking quality of services in integrated healthcare facilities.

## 1. Introduction

In modern and culturally diverse societies, patients have the opportunity to benefit from different approaches to healthcare. One of these approaches is based on integrating conventional Western medicine with different modalities of complementary and alternative medicine (CAM). This approach is known as “integrative medicine” [1, 2]. The concept of integrative medicine is a form of a holistic approach that is often used to cater to the different needs of patients. The concept goes beyond simply combining conventional medicine with CAM but also permits individualizing approaches to care delivery and incorporates the mid, body, spirituality, and a sense of belonging to the community [1–3].

Previous studies have shown that adopting an integrative medicine approach to patient care can bring important enhancements with regard to morbidity, mortality, quality of life, and short and long term costs of healthcare while caring for patients with different conditions like cancer [4], inflammatory [5], stroke [6], cardiovascular [7], and other diseases [8, 9].

Multihealthcare provider paradigms have been heavily promoted in contemporary healthcare systems. In these systems, the knowledge and expertise of pharmacists have been well recognized. Today, pharmacists are recognized as experts in providing pharmacotherapy and pharmaceutical care [10]. Therefore, pharmacists are assuming continuously evolving roles and responsibilities. Such expanding roles and responsibilities have recently been backed and advocated by professional organizations in different nations like the United States, United Kingdom, Australia, and Canada [11–15]. With the passage of time, more roles and responsibilities are expected to unfold for pharmacists. Because different CAM modalities are increasingly used in hospitalized patient settings as being either alternatives or complementary to conventional therapies [15, 16], pharmacists and other healthcare providers are expected to provide larger volumes of healthcare services to patients receiving such modalities. As a result, integrative medicine is being taught to healthcare professionals including pharmacists and physicians [17–20].

In practice, the concept of pharmaceutical care includes provision of direct services to patients like taking history, education/counselling, designing therapeutic plans, documentation, following up with patients, and collaborations with other healthcare providers [21]. In integrative medicine paradigms, pharmacists can provide many services that might include designing and implementing therapeutic plans, ordering laboratory tests, screening for, identifying, explaining, and resolving adverse reactions, interactions, ineffective therapies, and counselling/educating patients how to benefit the most out of their therapies which might include medications and CAM modalities [15, 16, 22].

In modern healthcare delivery paradigms, the focus has been shifted from provision of larger volumes of care to provision of higher quality care [23]. Recently, measuring and benchmarking provision of healthcare services and outcomes achieved through the efforts of healthcare providers has drawn a considerable attention [23–26]. As a result, different associations have developed quality measures that can be used to quantify and/or benchmark performance of healthcare providers across the continuum of care [23, 27]. To serve this purpose, key quality indicators are often developed for capturing and measuring performance of healthcare providers and quality of services provided. As efforts are surmounting to enhance healthcare delivery in healthcare facilities employing integrative medicine, measuring quality becomes imperative.

Recently, Alhusein and Watson narrated quality indicators with regard to community pharmacy services [28]. Boulkedid et al. conducted a systematic review on the use of the Delphi method for selecting quality indicators of healthcare [29]. However, the review focused on using and reporting the Delphi technique. Despite the importance of measuring the impact of pharmacists in integrative medicine, little was narrated on what activities and services can be used as quality indicators to capture the impact of pharmacists in integrative healthcare. To this date, there has been no comprehensive review summarizing the published literature on what activities and services that can be used to measure the impact of pharmacists in integrative medicine.

This scoping review was conducted to (a) identify, (b) describe, and (c) summarize different studies in which the Delphi technique was used to develop quality indicators of pharmaceutical care. The study also aimed to identify activities and services that could be used to capture the impact of pharmacist in integrative medicine. It is noteworthy mentioning that quality indicators are often developed using formal consensus technique. Because the Delphi technique is one of the most frequently used formal consensus techniques, this scoping review focused on studies that used the Delphi technique to develop quality indicators.

## 2. Methods

### 2.1. Study Design

This scoping review is reported in compliance with the Preferred Reporting Items for Systematic Reviews and Meta-Analyses statement for scoping reviews (PRISMA-ScR) [30]. Compliance to the PRISMA-ScR statement is shown in Supplementary [Supplementary-material supplementary-material-1]. The study protocol was informed by previous scoping reviews [28, 29, 31].

### 2.2. Literature Search Strategy

To identify studies reporting on different activities and services that could serve as quality indicators to capture the impact pharmacists in integrative healthcare facilities, a literature search and review was performed. The search was conducted using the following databases: EMBASE through PubMed, MEDLINE, COCHRANE, CINAHL hosted by EBESCO, and SCOPUS. The MeSH terms related to “quality indicators” and “Delphi technique” were mainly used. Related terms included “pharmacist,” “pharmacy,” “complementary therapies,” “complementary and alternative medicine,” “alternative medicine,” “integrative medicine,” “healthcare,” “delivery,” “provision,” “service,” “patients,” “hospitals,” “clinic,” “clinical,” “performance,” “indicators,” “standard,” “benchmark,” “metric,” “system,” “quality,” “evaluation,” “assessment,” and “review.” We used the Boolean operators “AND” and “OR” to combine terms. The search strategy was customized for each database. The literature was searched as late as February 02, 2020. A manual search of the references of the studies identified was also performed to identify potential studies that could be included. The search engine Google Scholar was also used to supplement the records identified through the databases. Titles and abstracts of the studies identified through the search were screened manually before deciding on the studies that would be fully reviewed. The search was made more thorough with the intention of including all potential activities and services that could be used as quality indicators because measuring quality indicators was relatively new to CAM practice.

### 2.3. Selection of Studies

The main investigator (PhD) who had prior knowledge and practical experience in performing literature search conducted the literature search. The search results were imported from the electronic databases as Research Information Systems (RIS) files and stored into EndNote (Clarivate Analytics, Philadelphia). Titles and abstracts were screened for eligibility and duplicates were removed. Duplicate records were removed using the “move references to trash” option in EndNote. The screening process was repeated trice to ensure reproducibility and another researcher verified the outcomes. Both researchers discussed discrepancies and resolved them by consensus. Studies that were finally selected for full text review were uploaded into EndNote using the “file attachment” option.

### 2.4. Inclusion Criteria

Documents were included if they were original articles reporting on development of quality indicators of pharmaceutical care or reporting on activities and services that could be used in capturing the impact of pharmacists in providing services relative to medications and CAM modalities that were developed using the Delphi technique. We did not restrict the search to country of origin, publication year, or publication status. Studies reporting mixed methods that included the Delphi technique were also included. Studies with mention of quality, pharmacists, CAM, and/or quality/performance indicators were given a priority for a full text review.

### 2.5. Exclusion Criteria

Studies reported in languages other than English were excluded. Additionally, studies were excluded when they were reported as poster presentations, commentaries, review articles, letters to the editor, and editorials, reported on one aspect of quality or reported on the quality of implementing a software or robotics. The aim of this scoping review was to identify quality indicators used to measure holistic services which often come in a set including services relevant to provision of CAM modalities. Again, studies that were not related to quality and/or performance indicators were excluded.

### 2.6. Extraction of Items

The form used to collect the data was created in an Excel spreadsheet (Microsoft Inc.). The data collection form is presented in Supplementary [Supplementary-material supplementary-material-1]. Documents selected were reviewed in full text by the main investigator (PhD). Text describing an activity or a service that potentially could be used as a quality indicator within the full text of each document was highlighted using the highlight text tool in Adobe Acrobat Pro (Adobe Inc., California). Sticky notes were added when and wherever needed.

Items were extracted by the main investigator directly into the data collection form. To ensure reproducibility, the process was repeated trice. Data collected from the literature were reviewed by another researcher independently. Whenever discrepancies were identified, researchers discussed these discrepancies and resolved them through consensus.

The data collected included author(s), publication year, setting and/or country, objectives of the study, study design, study participants, data collection, analysis, main results, and source of funding. The data extraction form is presented in Supplementary [Supplementary-material supplementary-material-1]. Because of their heterogeneous natures, findings of this scoping review are presented as narrative synthesis.

## 3. Results

### 3.1. Literature Search and Review

The literature search resulted in a total of 587 documents. Retrieved documents were screened for eligibility using titles and abstracts after removing the duplicates. Finally, data were extracted from 31 documents. Of those, 24 (77.4%) were related to provision of pharmaceutical services relevant to medications and 7 (22.6%) were related to provision of care using CAM modalities. The PRISMA flow diagram of the study selection is shown in Supplementary [Supplementary-material supplementary-material-1] [32].

### 3.2. Characteristics of the Selected Studies

**Figure 1:**
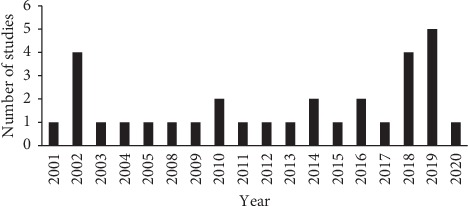
Number of studies published per year reporting on development of quality indicators selected for this scoping review.

The studies included were published between 2001 and 2020. Most of the studies (67.7%) were published in 2010 and beyond (Figure 1).

**Figure 2:**
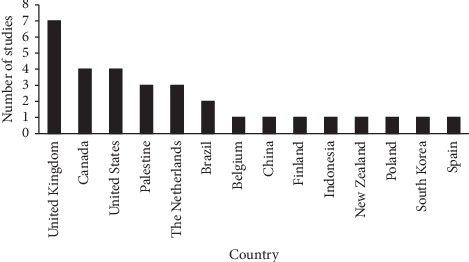
Country in which the study was conducted.

Almost half of the studies (48.4%) originated from the United States, United Kingdom, and Canada (Figure 2).

**Figure 3:**
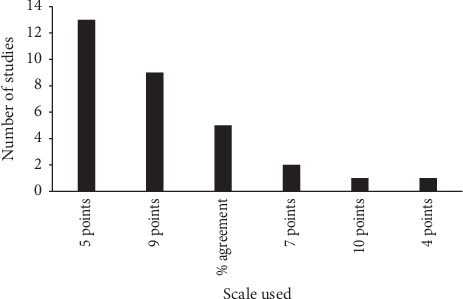
Scales used in the analysis of the votes of the participants in the selected studies.

The rest of the studies originated from Palestine, The Netherlands, Brazil, Belgium, China, Finland, Indonesia, New Zealand, Poland, South Korea, and Spain. The Likert-scale used to collect the votes of the panelists ranged from 4 to 10 points. About 42% of the studies used a 5-point Likert-scale (Figure 3).

**Figure 4:**
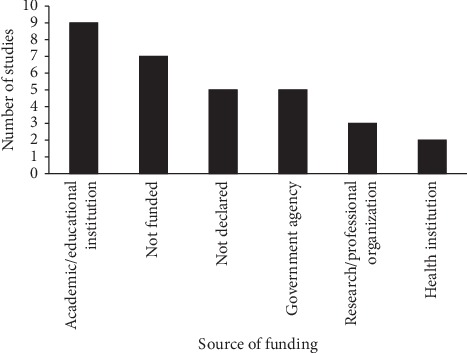
Fund sources of the studies selected for this review.

About 29% of the studies selected were funded by an academic or educational institution (Figure 4).

Table 1 shows a summary of the studies included in this scoping review. Additional information on the studies included is shown in Supplementary [Supplementary-material supplementary-material-1].

### 3.3. Development of Quality Indicators for Pharmaceutical Services Relevant to Medications

Fernandes et al. conducted a study to develop performance indicators to improve clinical pharmacy practice and patient care in Canada [26]. A working group of frontline clinical and hospital pharmacists from all over Canada systematically developed a comprehensive list of potential performance indicators. Three authors conducted a comprehensive literature review to create an inventory of candidate performance indicators. The crude performance indicators were rated against a list of 11 ideal attribute criteria. Three authors extracted 8 thematic critical activity areas that the final list should contain. A modified Delphi technique of three rounds with an in-person meeting that was held between the Delphi rounds 2 and 3 was followed to develop the final list. The Delphi rounds were completed by 26 pharmacists who had experience of 6–10 (*n* = 2), 11–15 (*n* = 5), and greater than 15 years (*n* = 19). The final list contained 8 performance indicators grouped into 6 categories: discharge medication reconciliation (*n* = 1), admission medication reconciliation and best possible medication history (*n* = 1), interprofessional patient care rounds (*n* = 1), pharmaceutical care (*n* = 2), bundle of critical activity areas (*n* = 1), and patient education/discharge counselling (*n* = 2).

In Palestine, a study was conducted to develop key performance indicators to capture the impact of pharmacists in providing pharmaceutical services [23]. Potential performance indicators were collected into an initial inventory list through literature search and review. To supplement the list identified through the literature search, neurologists (*n* = 2), pharmacists (*n* = 6), nurses (*n* = 3), and patients (*n* = 2) were also interviewed. A final core list of consensus-based key performance indicators was developed though a three-round Delphi technique among panelists (*n* = 40). Potential performance indicators in the initial inventory list were voted in the first Delphi round. The voting process in the first Delphi round resulted in 41 services being rated as useful by 60% and more of the panelists. Upon recommendations of the panelists, services were grouped and further 2 iterative Delphi rounds were then conducted to develop the final consensus-based core list. The final core list contained 8 performance indicators that were grouped into the thematic areas of pharmaceutical care (*n* = 3), patient education/counselling/reconciliation (*n* = 2), medication reconciliation and best possible medication history (*n* = 1), interprofessional patient care (*n* = 1), and competence and performance efficiency/patient satisfaction (*n* = 1).

Krzyżaniak et al. conducted a study in Poland to identify and develop a core list of essential roles and activities that could serve as performance indicators of pharmacy services in Poland [33]. A literature review was conducted to identify potential performance indicators. Authors consulted healthcare providers to determine if the potential performance indicators were suitable for the Polish pharmacy practice. A panel of 13 members which included pharmacist/director of pharmacy (*n* = 9), pharmacists in academia (*n* = 2), neonatologist/doctor (*n* = 1), and nurse/midwife (*n* = 1) was presented with 25 potential performance indicators grouped into structure (*n* = 9), process (*n* = 9), and outcome (*n* = 7) indicators. A two-round iterative Delphi technique was followed using an online questionnaire to achieve consensus on the final core list of performance indicators. The final core list contained 23 performance indicators grouped into structure (*n* = 9), process (*n* = 9), and outcome (*n* = 5) indicators.

In Belgium, Cillis et al. conducted a study for developing and validating a benchmarking tool to measure clinical pharmacy activities [34]. Authors conducted a narrative literature review to identify and collect potential performance indicators. Two focus groups were held to refine the list of collected performance indicators and a three-round Delphi technique was followed to achieve consensus on a final core list. The panelists who participated in the study were pharmacists who provided services in geriatrics, surgery, ICU, cystic fibrosis, nonpatient-centered activities, anticoagulation, antibiotic therapy management group, and nutrition areas. The final core list contained 10 quality indicators grouped into 6 areas: medication reconciliation at admission, patient monitoring, information provided to the healthcare team, patient education, discharge and transfer medication counselling, and adverse drug reaction monitoring.

Ng and Harrison conducted a study in New Zealand in which they used surveys and a Delphi technique in the identification of a list of key performance indicators that could be measured to demonstrate contributions of pharmacists in patient care [35]. In their study, potential items were collected from the literature and presented to the panelists. The panelists rated the items in the Delphi technique. Respondents (*n* = 44) were chief medical officers (*n* = 12), director of nursing (*n* = 5), chief pharmacists (*n* = 15), quality and risk managers (*n* = 8), and senior management team members (*n* = 4). Performance indicators were ranked by scores of relevance and measurability. Indicators included the following: chart review, medication reconciliation, prescribing errors, clinical pharmacy interventions, medication card provision, correct pediatric medication orders, adjustment or review of toxic or subtherapeutic doses, patient reviews, provision of written information to patients, and patient counselling.

Lima et al. conducted a study to develop and validate performance indicators for services related to medication management in Brazil [36]. The methodological study combined the Delphi technique with quantitative approaches to develop and validate the performance indicators. Potential performance indicators were identified by a working group which included university professors and researchers in clinical pharmacy (*n* = 2), doctoral students (*n* = 2), and clinical pharmacists (*n* = 4). The working group identified 7 performance indicators from the literature using their own professional experience. The performance indicators identified by the working group were rated for 7 attributes using a Likert-scale of 5 points in 2 iterative Delphi technique rounds among a panel of experts (*n* = 11). Views of practicing pharmacists (*n* = 82) were also sought using an online questionnaire for constructing validity and reliability. Content and construct validity were acceptable for 6 performance indicators which were grouped in the following categories: pharmaceutical consultation, interventions accepted by the prescriber, therapy problems solved, assessment of patient clinical status, satisfaction of the patient, and quality of life of the patient.

De Bie et al. conducted a study to develop a system of quality indicators for pharmaceutical care in The Netherlands [37]. The study used a literature review with a two-round Delphi technique which was then followed by a field test. A thorough literature review was conducted to compose an initial list of indicators. A two-round Delphi technique was followed to develop and validate the final list of indicators. Indicators in the final list were used to collect data from 30 pharmacies. The first Delphi round was completed by 16 panelists who were pharmacists (*n* = 14) and pharmacy technicians (*n* = 2) and the second round was completed by 151 pharmacists. The final list consisted of 42 quality indicators grouped into 6 categories: patient counselling (*n* = 6), clinical risk management (*n* = 10), compounding (*n* = 7), dispensing of medication (*n* = 3), monitoring of medication use (*n* = 11), and quality management (*n* = 5).

Grey et al. conducted a study to seek confirmation of stakeholders and rank in order of importance a list of characteristics of good pharmaceutical care in the United Kingdom [38]. The study was conducted in phases. In the first phase, a postal questionnaire was sent to community pharmacists and dispensing doctors to identify activities that could be considered characteristics of good pharmaceutical care. In-depth case studies were conducted with community pharmacists (*n* = 3) and dispensing doctors (*n* = 4) and analyzed thematically. A two-round iterative Delphi technique was followed among a panel to achieve consensus on a final list of characteristics of good pharmaceutical care in the United Kingdom. The panelists who voted in the first Delphi round were dispensing doctors (*n* = 3), community pharmacists (*n* = 8), community pharmacy dispensing assistants (*n* = 2), community pharmacy board members (*n* = 1), large chain community pharmacy executives (*n* = 2), and laypersons (*n* = 7). The final list contained 23 characteristics of good pharmaceutical care grouped into 4 categories: patient safety dispensing (*n* = 6), patient–provider interaction (*n* = 6), workplace culture (*n* = 7), and public health (*n* = 4).

Clay et al. conducted a study in the United States to develop a checklist of pharmacist interventions while providing patient care services [39]. In this study, a list of items was collected through expert group meetings, literature review, and refinement of the items through iterative rounds including face-to-face meetings, conference calls, and receiving public comments. The final list received input from more than 200 stakeholders over a period of 4 years. Using a modified Delphi technique, the final list contained 9 critical components: replicability, patient population, patient and other data sources, environment, delivery, frequency and duration, pharmacist role and responsibility, attribution, and unique attributes.

Quality indicators of preventing medication related morbidity were the subject of different studies conducted in the United States [40], United Kingdom [41, 42], and Canada [43]. Morris et al. described the process of developing and validating a series of indicators that could be used to prevent drug-related morbidity [42]. The indicators were developed using a two-round Delphi technique among panelists (*n* = 16) who were general practitioners (*n* = 6) and pharmacists (*n* = 10). The indicators selected for the study were validated in a preliminary step. A two-round Delphi technique was followed among the panelists to develop the final list. In the final list, there were 29 indicators; of those, 19 were originally developed for practice in the United States practice and 10 were generated by the panelists for practice in the United Kingdom. In their work, Morris and Cantrill assessed a series of preventable drug-related morbidity indicators used in the United States for applicability in the United Kingdom after transferring patients from the United States to the United Kingdom healthcare facilities [41]. A two-round Delphi technique was followed among a panel (*n* = 16) of experts and preventable drug-related morbidity indicators were taken from previous studies and presented to the panelists for consensus. The final list contained 19 indicators of possible preventable drug-related morbidity situations that could be applied in the United Kingdom healthcare settings. Robertson and MacKinnon conducted a study in Canada to develop clinical indicators of preventable drug-related morbidity in older adults [43]. The study used a two-round Delphi technique followed by a focus group. Two separate panels of specialists were composed for the study. The first panel included geriatricians (*n* = 6) and the second panel included clinical pharmacologists (*n* = 6). Indicators were developed using the Delphi technique. General practitioners (*n* = 12) participated in a focus group to assess the applicability of the indicators for practice in Canada. The final list included 52 clinical indicators of preventable drug-related morbidity that can be applied and used in practice in Canada. Mackinnon and Hepler conducted a study to develop a list of clinical indicators of preventable drug-related morbidity [40]. An initial list of potential indicators was composed following a literature review. The literature was reviewed to identify scenarios that represented potential outcomes and patterns of care that were thought to be possible preventable drug-related morbidity situations in older adults. A two-round Delphi technique was then followed among a panel of physicians (*n* = 6) and a clinical pharmacist (*n* = 1) to develop the final list of clinical indicators of preventable drug-related morbidity in older adults. The final list contained 52 scenarios representing possible preventable drug-related morbidity situations in older adults.

In the United States, Pyne et al. conducted a study to develop a valid and usable list of quality indicators to detect and treat depression in patients [44]. The literature was reviewed to collect potential quality indicators for detection of depression in patients. A modified Delphi technique was followed to develop the final list of quality indicators in detecting and treating depression in patients. The panelists were physicians (*n* = 6), psychiatrists (*n* = 4), and a clinical pharmacist (*n* = 1). The final list contained 59 quality indicators grouped into 6 categories: general indicators for depression treatment in patients, bereavement, substance abuse, viral infections, cognitive impairment, and mental health drug interactions.

Currie et al. conducted a study in the United States with the aim of developing guidelines to document elements needed to record care provided by pharmacists to allow assessment of quality of care [45]. The literature was reviewed and an initial list was compiled. A group of pharmacists validated the list. A three-round Delphi technique followed by group meetings was conducted among the panelists to achieve consensus on the final list. The panelists were pharmacists (*n* = 9) and other experts (*n* = 8). The final list contained elements of documentation as a tool to evaluate documentations (*n* = 14). The study concluded that the list might serve as a tool to assess the quality of care provided and documented by pharmacists.

In the United States, Malone et al. conducted a study to develop a list of clinically important drug-drug interactions that could be encountered and detected by pharmacist through a computerized pharmacy system [46]. The literature was reviewed. Potential items collected were presented to the panelists in a modified Delphi technique. The panelists were physicians (*n* = 2), clinical pharmacists (*n* = 2), and one expert in drug-drug interactions (*n* = 1). The final list consisted of 56 drug-drug interactions. Consensus was achieved among the panelists to consider 25 drug-drug interactions as clinically important.

Puumalainen et al. conducted a study in Finland to develop validated and easy to use patient counselling quality assurance tool for pharmacists. For this study two separate panels were recruited. The first panel included practicing pharmacists (*n* = 10) and the second panel included academic and professional experts (*n* = 10). The panelists developed indicators for the tool. The Delphi technique was followed among the panelists to develop the final tool. The final tool contained 16 indicators grouped into 3 quality groups relevant to patient (*n* = 4), process (*n* = 6), and learning and innovations (*n* = 6).

In another study conducted by Bowie et al. in the United Kingdom a mixed method of small groups, workshops, modified Delphi technique, and interviews was used in the development and prioritization of a list of safety-critical issues to be addressed in the first period of general practice training [47]. In this study, items and themes were generated and refined using a mixed method which included iterations in small group meetings, a modified Delphi technique, and interviews. The study participants were general practitioner educators (*n* = 127) and specialty trainees (*n* = 9). The final list contained 47 safety-critical issues organized under 14 themes: prescribing safely (*n* = 6), dealing with medical emergency (*n* = 4), specific clinical management (*n* = 1), dealing effectively with results of investigation requests (*n* = 2), patient referrals (*n* = 4), effective and safe communication (*n* = 3), consulting safely (*n* = 3), ensuring confidentiality (*n* = 2), awareness of the implications of poor record keeping (*n* = 5), raising awareness of personal responsibility (*n* = 4), dealing with child protection issues (*n* = 3), enhancing personal safety (*n* = 3), emphasizing the importance of the learning environment (*n* = 4), and safe use of practice computerized systems (*n* = 3).

Fernández-Llamazares et al. conducted a study in Spain with the aim of designing and achieving consensus on a pediatric pharmaceutical care model [48]. In their study, a two-round Delphi technique was used. The panelists were experts (*n* = 50) from 20 different hospitals. Items were developed using an iterative process. A two-round Delphi technique was followed among the panelists to achieve consensus. The final model contained 39 items grouped used in basic validation (*n* = 17), intermediate level (*n* = 13), and advanced level (*n* = 9).

Floor-Schreudering et al. conducted a study in The Netherlands aiming at developing drug-drug interaction management guidelines to support healthcare professionals in clinical practice [49]. A two-round Delphi technique was employed in the study. A panel (*n* = 23) was voted in the Delphi rounds. The panelists included pharmacists, physicians, educators, and clinical pharmacologists. The panelists expressed their views and opinions on a list of potential items relevant to management of drug-drug interactions. The final list contained 15 elements in a standardized report which included quality of evidence for harm, level of evidence, pharmacological plausibility, seriousness, incidence of outcomes, clinical impact on the population, susceptibility factors, clinical impact on the patient, strength of recommendations, what to manage, when to start management, how to monitor, when to stop management, a set of communication tools, and a brief summary.

Tonna et al. conducted a study in the United Kingdom to develop guidelines to facilitate service redesign around pharmacist prescribing [50]. A two-round Delphi technique was used in the study. Statements were presented to the panelists in the two-round Delphi technique. The panel (*n* = 35) included directors of pharmacy (*n* = 4), directors of hospital services (*n* = 3), chairmen of area drug and therapeutics committee (*n* = 4), nonpharmacist authors of local nonmedical prescribing policy (*n* = 5), pharmacist authors of local nonmedical prescribing policy (*n* = 10), and pharmacist prescribers (*n* = 15). The final list contained 27 statements which were related to two domains: service development and pharmacist prescribing role development. Service development included succession planning (*n* = 8), multiprofessional working (*n* = 6), quality evaluation (*n* = 2), practice development (*n* = 2), and outcome measures (*n* = 1). Pharmacist prescribing role development included education (*n* = 7) and future orientation of service (*n* = 1).

In the United Kingdom, Aljamal et al. used a modified Delphi technique in a study with the aim of developing and examining appropriateness of indicators of medication reconciliation [51]. The panelists (*n* = 65) were hospital pharmacists with pharmacy degree only (*n* = 4), postgraduate diploma (*n* = 34), master's degree (*n* = 23), and other degrees (*n* = 4). An initial list of indicators was presented to the panelists. Consensus was achieved in a two-round Delphi technique. The final list contained 41 indicators grouped into collecting (*n* = 16), checking (*n* = 12), communicating (*n* = 7), and entire process (*n* = 6).

Satibi et al. conducted a study in Indonesia for the purpose of developing performance indicators to measure quality of pharmacy services [52]. The literature was reviewed and an initial list was compiled. A group of pharmacists validated the list. A three-round Delphi technique followed by group meetings was conducted among the panelists to achieve consensus on the final list. The panel (*n* = 15) included pharmacist practitioners at primary health centers (*n* = 12), representative of the regency health office (*n* = 3), and chairperson of the province health service quality agency (*n* = 1). The final list contained 26 indicators of drug management, 19 indicators of clinical pharmacy services, and 2 indicators of overall pharmacy performance.

In Brazil, Rocha et al. used a mixed method of iterations, meetings, and a Delphi technique in the development and validation of a tool to support pharmaceutical counselling of patients with regard to medications [53]. Iterations, repeated meetings, and Delphi technique rounds were used to develop and validate the tool that can be used to support pharmaceutical counselling of patients with regard to medications. The panel (*n* = 29) included pharmacists with basic pharmacy degree (*n* = 2), specialization course (*n* = 13), and master's or doctoral degree (*n* = 14). The final tools contained 3 components: suggestions for questions, dispensing process reasoning, and suggestions for counselling.

### 3.4. Quality Indicators for Services Relevant to CAM Modalities

Im et al. conducted a study in South Korea with the aim of developing an evaluative scale to measure the effects of horticultural therapy in practical settings [6]. The study used a mixed method of qualitative and quantitative study design with preliminary studies. Items collected from the interviews and the literature were presented to the panelists in the Delphi technique. The study participants were horticultural therapists (*n* = 779) who answered open-end questionnaire. In-depth interviews were conducted with horticultural therapists (*n* = 20). Panelists (*n* = 24) participated in the Delphi technique. The final list of effects of horticultural therapy was categorized into 4 aspects: physical (*n* = 27 items), cognitive (*n* = 25 items), psychoemotional (*n* = 24 items), and social (*n* = 22).

In The Netherlands, van Overveld et al. conducted a study for the development of multidisciplinary quality indicators for measurement of quality of integrated oncological care [54]. In their study, the literature was reviewed and a modified Delphi technique was used. Two separate panels were recruited in this study. The first panel included medical specialists (*n* = 18) and second panel included allied health practitioners (*n* = 11). Items collected from the interviews and the literature were presented to the panelists in the Delphi technique. The final list contained structure, process, and outcome indicators. The list of medical specialists contained 5 outcome and 13 process indicators. The list of the allied health professionals contained 3 structure, 19 process, and 5 outcome indicators.

In Palestine, Shawahna et al. conducted a study to develop a list of harms and benefits of using fenugreek for breastfeeding women that need to be discussed during clinical consultations [55]. Literature review and interviews followed by a two-round Delphi technique were used. In this study, two separate panels of healthcare providers (*n* = 56) and breastfeeding women (*n* = 65) were composed. Potential items were collected from the literature and interviews and presented to the panelists. The panelists rated the items in a Delphi technique. The final list contained 34 items grouped into harms (*n* = 21) and benefits (*n* = 13). In another study, Shawahna and Al-Atrash developed a list of knowledge items that healthcare providers and CAM practitioners need to know on the benefits of exercise as a CAM modality in cancer [56]. In this study, interviews, literature review, and a two-round Delphi technique were used. Items collected from the interviews and the literature were presented to the panelists in two-round Delphi technique. The panel (*n* = 65) included healthcare providers and CAM practitioners. The final list contained 45 items grouped into 6 categories: general items (*n* = 9), effects on the immune system (*n* = 16), anticancer treatment (*n* = 12), metastasis (*n* = 3), tumor metabolism (*n* = 3), and release of myokines (*n* = 2).

Richardson conducted a study in the United Kingdom to develop indicators for referral to an outpatient service providing CAM modalities by considering the research evidence for the effectiveness of these modalities [57]. The study employed a survey which was then followed by a modified Delphi technique. General practitioners were surveyed for their opinions with regard to referring patients to outpatient services providing CAM modalities. A modified Delphi technique was used to develop indicators for referral to an outpatient service providing CAM modalities. General practitioners (*n* = 71) were surveyed and healthcare professional panelists (*n* = 27) took part in the modified Delphi technique. The panelists agreed on developing indicators for referral to services providing CAM modalities like acupuncture, homeopathy, and osteopathy in conditions like allergic conditions (rheumatoid arthritis, osteoarthritis, asthma, chronic obstructive airways disease, and rhinitis), back pain, neurologic conditions, palliative care, irritable bowel syndrome and reflux oesophagitis, eczema, emotional disorders, eye and mouth disorders, prolapse/endometriosis/menstrual problems, headaches, stress/fatigue, insomnia, hypertension, skeletal problems, strokes, tinnitus, viral conditions, and common childhood disorders.

Byrne et al. conducted a study in Canada to develop core competencies in natural health products that future pharmacists should possess [58]. The study employed a modified Delphi technique. A list of potential competencies was compiled from previous qualitative and survey studies. A four-round Delphi technique was followed among the panelists to develop and achieve consensus on the final list. The panelists (*n* = 17) were pharmacy educators, academic administrators, and representatives from Canadian pharmacy organizations. All panelists had interest in natural health products. The final list contained competencies grouped into 3 areas: knowledge of natural products when providing pharmaceutical care, access to and critical appraisal of information sources, and provision of appropriate patient education on effects, adverse reactions, and interactions of natural health products.

Guangyi et al. conducted a study in China to develop a list of traditional Chinese medicine symptoms and signs for screening chronic low back pain [59]. In this study, a three-round Delphi technique was used. Items collected from the interviews and the literature were presented to the panelists in the Delphi technique. The panelists (*n* = 13) were experts in orthopedics, massage, and acupuncture. The final list contained 35 diagnostic characteristics grouped into pain characteristics (*n* = 8), associated factors (*n* = 11), and physical and tongue diagnostic expressions (*n* = 16).

### 3.5. Activities and Services That Could Be Used as Quality Indicators Identified from the Studies Reviewed

Table 2 lists 42 different activities and services that could be used as quality indicators identified from the studies included in this review. Activities and services were related to history taking, performing reconciliations, identifying and resolving therapy problems, providing collaborative care, designing care plans, optimal performance, and continuing education.

## 4. Discussion

Quality indicators in healthcare are often developed using qualitative and formal consensus techniques. Since its inceptions, the Delphi technique has evolved as one of the most frequently used formal consensus techniques in healthcare [60]. In this scoping review, quality indicators of pharmaceutical care in integrated healthcare facilities are appraised for the first time with special focus on those developed using the Delphi technique as a formal consensus method. The present scoping review synthesizes for the first time identifiable activities and services that can serve as quality indicators. This scoping review also sheds light on the different perspectives of using this formal approach in developing usable quality indicators in healthcare for the purpose of improving services provided by pharmacists in integrated healthcare facilities.

Using the search method adopted for this review, 31 studies were identified and included in the narrative analysis. These studies were published over a span of about 20 years (2001 to 2020). The studies included in this review originated from 14 different countries and used different definitions of consensus and scales to collect views of the panelists who participated in the development of quality indicators. In general, this scoping review demonstrated an increasing interest of quality indicators in healthcare.

It has been argued that quality improvement of services should be reflected in the context in which it is offered [23, 61, 62]. Studies included in this scoping review were conducted in different countries and settings, funded by different interested parties, included diverse populations of stakeholders, and were conducted over a long span of years. This might have an impact on their generalization and applicability on the international level. Therefore, it could be important to consider the context in which the different quality indicators were developed in the reviewed studies to inform decisions on their transferability to other healthcare settings in different countries. Another important factor to consider is the jurisdictions in which these quality indicators were developed and the ones to which they are intended to be transferred in the light of what services pharmacists are legally allowed to provide [63–65].

Overall, qualitative research methods and formal consensus techniques are preferred in developing quality indicators for use in healthcare [28]. These methods are often preferred in the absence of gold standards in what to consider as quality indicators relative to structure, process, and outcome in healthcare. In such cases, formal consensus methods provide useful means when the only alternatives are subjective or anecdotal [23, 56, 66]. As one of the most commonly used formal consensus methods, the Delphi technique might help reduce bias, impart transparency, add validity, and strengthen judgmental approaches use to develop concepts like quality indicators in healthcare [55, 67, 68]. It has been argued that stakeholders would be more interested in using quality indicators they agree with than quality indicators they do not agree with.

In the studies included, stakeholders who were involved in the development of quality indicators were from both genders, different age groups, had variable experience, and were from different specialty backgrounds. The panelists were pharmacists, pharmacy assistant, physicians, nurses, midwives, academicians, researchers, postgraduate students, quality, and risk managers. Involvement of those stakeholders is relevant, as those experts with such background are key healthcare providers and have interest in improving quality of healthcare. Diversifying involvement of stakeholders has been argued to add validity and strength to concepts developed through formal consensus techniques [69–71]. A part from interviews in the initial stages of collecting potential quality indicators, the present scoping review showed limited involvement of patients as panelists who were sought to vote on the inclusion or exclusion of quality indicators in the final lists of quality indicators. Findings of this review were consistent with those previously reported by Alhusein and Watson [28]. It is probably important for future studies to include patients as panelists who can actively participate in the voting process and selection of the consensus-based quality indicators because patients are the clientele and, in many healthcare systems, satisfaction of the clientele has been considered a high priority [72, 73]. It is noteworthy mentioning that priorities might change with time and hence quality indicators might need to be revisited periodically to keep up with changes in priorities and evolution of roles and services that could be played by pharmacists [40–42].

The activities and services identified from the studies included in this scoping review broadly cover the different aspects of pharmaceutical services that could be offered in healthcare facilities providing integrated care. Such services might reflect the continuously expanding roles and responsibilities of pharmacists relevant to provision of collaborative, direct, and proactive care to patients, counselling/educating patients on their diseases and therapeutic options, and resolving therapy related problems [15, 16, 21, 22].

The present scoping review demonstrated that little efforts were focused on developing quality indicators of pharmaceutical services and, especially, those relevant to CAM in healthcare facilities adopting an integrated paradigm of healthcare delivery. This scoping review stresses on the current need to direct future studies at developing quality indicators relevant to CAM provision in healthcare facilities adopting an integrated paradigm of healthcare delivery.

### 4.1. Limitations of the Study

The findings of this scoping review could be interpreted after carefully taking into account the following points. First, only articles published in English were included in this review. Restricting the search to works published in English could have excluded some interesting articles that might have been published in other languages than English. Second, this review was not systematic; rather, a scoping review was conducted. Systematic reviews of the literature have been advocated as they provide robust and reproducible results compared with other types of reviews. The decision not to use a systematic review was informed after considering the objectives of the study, nature of the research questions, the problem, intervention, comparison, outcome, study design (PICOS), scope, number, and nature of the documents to be included in the study [74–76]. Third, a narrative synthesis approach was adopted to present the results of the publications reviewed in this study. A part from the summary of the results provided in the results section, this scoping review did not provide a detailed analysis of the findings included in the original publications. Inclusion of such details could have gone beyond the scope of this review. Fourth, this scoping review did not include assessment of the scientific quality of the articles included in the narrative synthesis. The focus on this scoping review was quality indicators developed using the Delphi technique; however, quality indicators developed using other methods were not included. Finally, studies reporting measuring quality of healthcare using the developed quality indicators were not included in this scoping review.

## 5. Conclusions

Although there is an increasing interest in improving healthcare delivery, quality indicators of pharmaceutical services and those relevant to CAM provision in healthcare facilities adopting the integrated healthcare paradigm are still limited. Future studies are needed to develop validated quality indicators that could be successfully used in measuring and benchmarking quality of pharmaceutical and CAM services in integrated healthcare facilities.

## Figures and Tables

**Table 1:** Summary of the studies included in this scoping review (*n* = 31).

No.	Author(s)	Objectives of the study	Participants	Data collection	Main results
*Pharmaceutical services relevant to medications*					
1	Fernandes et al. [26]	To develop performance indicators to improve clinical pharmacy practice and patient care	The Delphi rounds were completed by 26 pharmacists: experience of 6–10 (*n* = 2), 11–15 (*n* = 5), and greater than 15 years (*n* = 19)	A working group of frontline clinical and hospital pharmacists from all over Canada systematically developed a comprehensive list of potential performance indicators. Three authors conducted a comprehensive literature review to create an inventory of candidate performance indicators. The crude performance indicators were rated against a list of 11 ideal attribute criteria. Three authors extracted 8 thematic critical activity areas that the final list should contain. A modified Delphi technique of three rounds with an in-person meeting was followed to develop the final list.	The final list contained 8 performance indicators grouped into 6 categories: discharge medication reconciliation, admission medication reconciliation and best possible medication history, interprofessional patient care rounds, pharmaceutical care, bundle of critical activity areas, and patient education/discharge counselling.
2	Shawahna [23]	Development of a core set of key performance indicators to measure impact of pharmacists in caring for patients	Pharmacists (*n* = 25), nurses (*n* = 4), physicians (*n* = 4), and doctorates (*n* = 7)	A formal consensus technique using the Delphi technique (literature search, interviews with 14 pharmacists, neurologists, nurses, and patients, and a three-round Delphi technique among 40 panelists).	The final core list contained 8 key performance indicators in the following thematic areas: pharmaceutical care (*n* = 3), patient education/counselling/reconciliation (*n* = 2), medication reconciliation and best possible medication history (*n* = 1), interprofessional patient care (*n* = 1), and competence and performance efficiency/patient satisfaction (*n* = 1).
3	Krzyżaniak et al. [33]	To identify and develop a core list of essential roles and activities that could serve as performance indicators of pharmacy services in Poland	Pharmacist/director of pharmacy (*n* = 9), pharmacists in academia (*n* = 2), neonatologist/doctor (*n* = 1), and nurse/midwife (*n* = 1)	A literature review was conducted. Healthcare providers were consulted for suitability of the potential indicators identified. A modified Delphi technique of two consecutive online rounds was conducted.	The final core list contained 23 performance indicators for quality pharmaceutical care grouped into structure (*n* = 9), process (*n* = 9), and outcome (*n* = 5) indicators.
4	Cillis et al. [34]	Developing and validating a benchmarking tool to measure clinical pharmacy activities	17 pharmacists who provided services in geriatrics, surgery, intensive care unit, cystic fibrosis, nonpatient-centered activities, anticoagulation, antibiotic therapy management group, and nutrition	A narrative literature review was conducted by the authors to identify and collect potential performance indicators. Two focus groups were held to refine the list of collected performance indicators. A three-round Delphi technique was followed to achieve consensus on a final core list.	The final core list contained 10 quality indicators grouped into 6 areas: medication reconciliation at admission, patient monitoring, information provided to the healthcare team, patient education, discharge and transfer medication counselling, and adverse drug reaction monitoring.
5	Ng and Harrison [35]	Identification of a list of key performance indicators that could be measured to demonstrate contributions of pharmacists in patient care	Respondents (*n* = 44) were chief medical officers (*n* = 12), director of nursing (*n* = 5), chief pharmacists (*n* = 15), quality and risk managers (*n* = 8), and senior management team members (*n* = 4).	Potential items were collected from the literature and presented to the panelists. The panelists rated the items in a Delphi technique.	Performance indicators were ranked by scores of relevance and measurability. Indicators included: chart review, medication reconciliation, prescribing errors, clinical pharmacy interventions, medication card provision, correct pediatric medication orders, adjustment or review of toxic or subtherapeutic doses, patient reviews, provision of written information to patients, and patient counselling.
6	Lima et al. [36]	Development of a set of key performance indicators for services related to medication management	The working group consisted of university professors and researchers in clinical pharmacy (*n* = *2*), doctoral students (*n* = *2*), and clinical pharmacists (*n* = *4*). The questionnaire was administered on pharmacists (*n* = 82).	Iterative rounds were conducted to identify potential performance indicators. The indicators identified were rated by experts for 7 attributes using a Likert-scale of 5 points in 2 iterative Delphi technique rounds. An online questionnaire was administered on 82 pharmacists.	The final core list contained 6 performance indicators grouped in the following categories: pharmaceutical consultation, interventions accepted by the prescriber, therapy problems solved, assessment of patient clinical status, satisfaction of the patient, and quality of life of the patient.
7	De Bie et al. [37]	To develop a system of quality indicators for pharmaceutical care	The first Delphi round was completed by 16 panelists and the second round was completed by 151 pharmacists.	A thorough literature review was conducted to compose an initial list of indicators. A two-round Delphi technique was followed to develop and validate the final list of indicators. Indicators in the final list were used to collect data from 30 pharmacies.	The final list consisted of 42 quality indicators grouped into 6 categories: patient counselling, clinical risk management, compounding, dispensing of medication, monitoring of medication use, and quality management.
8	Grey et al. [38]	To seek confirmation of stakeholders and rank in order of importance a list of characteristics of good pharmaceutical care	The first round was completed by 23 participants who were dispensing general practitioners or practice managers (*n* = 3), community pharmacists (*n* = 8), community pharmacy dispensing assistants (*n* = 2), community pharmacy board members (*n* = 1), large chain community pharmacy executives (*n* = 2), and laypersons (*n* = 7).	A postal questionnaire was sent to community pharmacists and dispensing doctors to identify characteristics of good pharmaceutical care. In-depth case studies of community pharmacists (*n* = 3) and dispensing doctors (*n* = 4) were conducted. A two-round Delphi technique was then followed to confirm and rank in order of importance a list of characteristics of good pharmaceutical care.	The final list contained 23 characteristics of good pharmaceutical care grouped into 4 categories: patient safety dispensing, patient–provider interaction, workplace culture, and public health.
9	Clay et al. [39]	Development of a checklist of pharmacist interventions while providing patient care services	The final list received input from more than 200 stakeholders over a period of 4 years.	A list of items was collected through expert group meetings, literature review, and refinement of the items through iterative rounds including face-to-face meetings, conference calls, and receiving public comments.	The final list contained 9 critical components: replicability, patient population, patient and other data sources, environment, delivery, frequency and duration, pharmacist role and responsibility, attribution, and unique attributes.
10	Richardson [57]	To develop indicators for referral to an outpatient service providing CAM modalities by considering the research evidence for the effectiveness of these modalities	General practitioners (*n* = 71) were surveyed and healthcare professional panelists (*n* = 27) took part in the modified Delphi technique.	General practitioners were surveyed for their opinions with regard to referring patients to outpatient services providing CAM modalities. A modified Delphi technique was used to develop indicators for referral to an outpatient service providing CAM modalities.	The panelists agreed on developing indicators for referral to services providing CAM modalities like acupuncture, homeopathy, and osteopathy in conditions like allergic conditions (rheumatoid arthritis, osteoarthritis, asthma, chronic obstructive airways disease, and rhinitis), back pain, neurologic conditions, palliative care, irritable bowel syndrome and reflux oesophagitis, eczema, emotional disorders, eye & mouth disorders, prolapse/endometriosis/menstrual problems, headaches, stress/fatigue, insomnia, hypertension, skeletal problems, strokes, tinnitus, viral conditions, and common childhood disorders.
11	Mackinnon and Hepler [40] (review other studies)	Developing a list of clinical indicators of preventable drug-related morbidity in older adults	The panelists were physicians (*n* = 6) and a clinical pharmacist (*n* = 1).	The literature was reviewed to identify scenarios that represented potential outcomes and patterns of care that were thought to be possible preventable drug-related morbidity situations in older adults. A modified Delphi technique was followed among the panelists to develop the final list of clinical indicators of preventable drug-related morbidity in older adults.	The panelists agreed on 52 scenarios representing possible preventable drug-related morbidity situations in older adults.
12	Pyne et al. [44]	To develop a valid and usable list of quality indicators to detect and treat depression in patients	The panelists were physicians (*n* = 6), psychiatrists (*n* = 4), and a clinical pharmacist (*n* = 1).	The literature was reviewed to collect potential quality indicators for detection of depression in patients. A modified Delphi technique was followed to develop the final list of quality indicators in detecting and treating depression in patients.	The final list contained 59 quality indicators grouped into 6 categories: general indicators for depression treatment in patients, bereavement, substance abuse, viral infections, cognitive impairment, and mental health drug interactions.
13	Morris and Cantrill [41]	To assess if a series of preventable drug-related morbidity indicators used in the United States were applicable to the United Kingdom after transferring from the United States to the United Kingdom healthcare facilities	A panel of 16 members: general practitioners (*n* = 6) and primary care pharmacists (*n* = 10)	Preventable drug-related morbidity indicators were taken from previous studies and presented to the panelists for consensus.	The final list contained 19 indicators of possible preventable drug-related morbidity situations in older adults in the United Kingdom healthcare settings.
14	Morris et al. [42]	Description of the process of developing and validating a series of indicators that could be used to prevent drug-related morbidity	General practitioners (*n* = 6) and pharmacists (*n* = 10)	Indicators selected were validated in a preliminary step. A two-round Delphi technique was followed among the panelists to develop the final list.	The final list contained 29 indicators. Of those, 19 were originally developed in the United States practice and 10 were generated by the panelists for the United Kingdom practice.
15	Robertson and MacKinnon [43]	Development of clinical indicators of preventable drug-related morbidity in older adults	Two separate specialists panels: geriatricians (*n* = 6) and clinical pharmacologists (*n* = 6); general practitioners (*n* = 12) participated in the focus group.	The Delphi technique was followed in 2 separate specialist panels to develop and achieve consensus on the clinical indicators. General practitioners participated in the focus group to assess the applicability of the indicators in Canada practice.	The final list contained 52 clinical indicators of preventable drug-related morbidity in older adults that can be applied to Canada practice.
16	Currie et al. [45]	Development of guidelines to document elements needed to record care provided by pharmacists to allow assessment of quality of care	Pharmacists (*n* = 9) and experts (*n* = 8)	The literature was reviewed and an initial list was compiled. A group of pharmacists validated the list. A three-round Delphi technique followed by group meetings was conducted among the panelists to achieve consensus on the final list.	The final list contained elements of documentation as a tool to evaluate documentations (*n* = 14). This list might serve as a tool to assess the quality of care provided and documented by pharmacists.
17	Malone et al. [46]	Development of a list of clinically important drug-drug interactions that could be encountered and detected by pharmacist through a computerized pharmacy system.	The panelists were physicians (*n* = 2), clinical pharmacists (*n* = 2), and one expert in drug-drug interactions (*n* = 1).	The literature was reviewed.	The final list consisted of 56 drug-drug interactions. Consensus was achieved to consider 25 drug-drug interactions as clinically important.
18	Puumalainen et al.	Development of a validated and easy to use patient counselling quality assurance tool for pharmacists	Two separate panels: practicing pharmacists (*n* = 10) and academic and professional experts (*n* = 10)	The panelists developed indicators for the tool. The Delphi technique was followed among the panelists to develop the final tool.	The final tool contained 16 indicators grouped into 3 quality groups relevant to patient (*n* = 4), process (*n* = 6), and learning and innovations (*n* = 6).
19	Byrne et al. [58]	Developing core competencies in natural health products that future pharmacists should possess	The panelists (*n* = 17) were pharmacy educators, academic administrators, and representatives from Canadian pharmacy organizations. All panelists had interest in natural health products.	A list of potential competencies was compiled from previous qualitative and survey studies. A four-round Delphi technique was followed among the panelists to develop and achieve consensus on the final list.	The final list contained competencies grouped into 3 areas: knowledge of natural products when providing pharmaceutical care, access to and critical appraisal of information sources, and provision of appropriate patient education on effects, adverse reactions, and interactions of natural health products.
20	Bowie et al. [47]	Development and prioritization of a list of safety-critical issues to be addressed in the first period of general practice training	General practitioner educators (*n* = 127) and specialty trainees (*n* = 9).	Items and themes were generated and refined using a mixed method which included iterations in small group meetings, a modified Delphi technique, and interviews.	The final list contained 47 safety-critical issues organized under 14 themes: prescribing safely (*n* = 6), dealing with medical emergency (*n* = 4), specific clinical management (*n* = 1), dealing effectively with results of investigation requests (*n* = 2), patient referrals (*n* = 4), effective & safe communication (*n* = 3), consulting safely (*n* = 3), ensuring confidentiality (*n* = 2), awareness of the implications of poor record keeping (*n* = 5), raising awareness of personal responsibility (*n* = 4), dealing with child protection issues (*n* = 3), enhancing personal safety (*n* = 3), emphasizing the importance of the learning environment (*n* = 4), and safe use of practice computerized systems (*n* = 3).
21	Fernandez-Llamazares et al. [48]	Designing and achieving consensus on a pediatric pharmaceutical care model	A panel of experts (*n* = 50) from 20 different hospitals	Items were developed using an iterative process. A two-round Delphi technique was followed among the panelists to achieve consensus.	The final model contained 39 items grouped used in basic validation (*n* = 17), intermediate level (*n* = 13), and advanced level (*n* = 9).
22	Floor-Schreudering et al. [49]	Development of drug-drug interaction management guidelines to support healthcare professionals in clinical practice	A panel (*n* = 23) was voted in the Delphi rounds. The panelists included pharmacists, physicians, educators, and clinical pharmacologists.	The panelists expressed their views and opinions on a list of potential items relevant to management of drug-drug interactions.	The final list contained 15 elements in a standardized report which included quality of evidence for harm, level of evidence, pharmacological plausibility, seriousness, incidence of outcomes, clinical impact on the population, susceptibility factors, clinical impact on the patient, strength of recommendations, what to manage, when to start management, how to monitor, when to stop management, a set of communication tools, and a brief summary.
23	Tonna et al. [50]	Development of guidelines to facilitate service redesign around pharmacist prescribing	A panel (*n* = 35) included directors of pharmacy (*n* = 4), directors of hospital services (*n* = 3), chairmen of area drug and therapeutics committee (*n* = 4), nonpharmacist authors of local nonmedical prescribing policy (*n* = 5), pharmacist authors of local nonmedical prescribing policy (*n* = 10), and pharmacist prescribers (*n* = 15).	Statements were presented to the panelists in the two-round Delphi technique.	The final list contained 27 statements which were related to two domains: service development and pharmacist prescribing role development. Service development included succession planning (*n* = 8), multiprofessional working (*n* = 6), quality evaluation (*n* = 2), practice development (*n* = 2), and outcome measures (*n* = 1). Pharmacist prescribing role development included education (*n* = 7) and future orientation of service (*n* = 1).
24	Aljamal et al. [51]	Development and examination of appropriateness of indicators of medication reconciliation	A panel (*n* = 65) contained hospital pharmacists with pharmacy degree only (*n* = 4), postgraduate diploma (*n* = 34), master's degree (*n* = 23), and other degrees (*n* = 4).	An initial list of indicators was presented to the panelists. Consensus was achieved in a two-round Delphi technique.	The final list contained 41 indicators grouped into collecting (*n* = 16), checking (*n* = 12), communicating (*n* = 7), and entire process (*n* = 6).
25	Satibi et al. [52]	Development of performance indicators to measure quality of pharmacy services	The panel (*n* = 15) included pharmacist practitioners at primary health centers (*n* = 12), representative of the regency health office (*n* = 3), and chairperson of the province health service quality agency (*n* = 1).	The literature was reviewed and an initial list was compiled. A group of pharmacists validated the list. A three-round Delphi technique followed by group meetings was conducted among the panelists to achieve consensus on the final list.	The final list contained 26 indicators of drug management, 19 indicators of clinical pharmacy services, and 2 indicators of overall pharmacy performance.
26	Rocha et al. [53]	Development and validation of a tool to support pharmaceutical counselling of patients with regard to medications	The panel (*n* = 29) included pharmacists with basic pharmacy degree (*n* = 2), specialization course (*n* = 13), and master's or doctoral degree (*n* = 14).	Iterations, repeated meetings, and Delphi technique rounds were used to develop and validate the tool that can be used to support pharmaceutical counselling of patients with regard to medications.	The final tools contained 3 components: suggestions for questions, dispensing process reasoning, and suggestions for counselling.

*Pharmaceutical services relevant to CAM*					

1	Im et al. [6]	Development of an evaluative scale to measure the effects of horticultural therapy in practical settings	Horticultural therapists (*n* = 779) answered open-end questionnaire. In-depth interviews were conducted with horticultural therapists (*n* = 20). Panelists (*n* = 24) participated in the Delphi technique.	Items collected from the interviews and the literature were presented to the panelists in the Delphi technique.	The final list of effects of horticultural therapy was categorized into 4 aspects: physical (*n* = 27 items), cognitive (*n* = 25 items), psychoemotional (*n* = 24 items), and social (*n* = 22).
2	van Overveld et al. [54]	Development of multidisciplinary quality indicators for measurement of quality of integrated oncological care	Two separate panels: medical specialists (*n* = 18) and allied health practitioners (*n* = 11)	Items collected from the interviews and the literature were presented to the panelists in the Delphi technique.	The final list contained structure, process, and outcome indicators. The list of medical specialists contained 5 outcome and 13 process indicators. The list of the allied health professionals contained 3 structure, 19 process, and 5 outcome indicators.
3	Shawahna et al. [55]	Development of a list of using harms and benefits of using fenugreek for breastfeeding women that need to be discussed during clinical consultations	Two separate panels of healthcare providers (*n* = 56) and breastfeeding women (*n* = 65)	Potential items were collected from the literature and interviews and presented to the panelists. The panelists rated the items in a Delphi technique.	The final list contained 34 items grouped into harms (*n* = 21) and benefits (*n* = 13).
4	Shawahna and Al-Atrash [56]	Development of a list of knowledge items that healthcare providers and CAM practitioners need to know on the benefits of exercise as a CAM modality in cancer	The panel (*n* = 65) included healthcare providers and CAM practitioners.	Items collected from the interviews and the literature were presented to the panelists in two-round Delphi technique.	The final list contained 45 items grouped into 6 categories: general items (*n* = 9), effects on the immune system (*n* = 16), anticancer treatment (*n* = 12), metastasis (*n* = 3), tumor metabolism (*n* = 3), and release of myokines (*n* = 2).
5	Guangyi et al. [59]	Development of a list of traditional Chinese medicine symptoms and signs for screening chronic low back pain	Panelists (*n* = 13) were experts in orthopedics, massage, and acupuncture.	Items collected from the interviews and the literature were presented to the panelists in the Delphi technique.	The final list contained 35 diagnostic characteristics grouped into pain characteristics (*n* = 8), associated factors (*n* = 11), and physical and tongue diagnostic expressions (*n* = 16).

CAM: complementary and alternative medicine.

**Table 2:** Activities and services that could be used as quality indicators of pharmaceutical services relevant to medications and CAM in integrative medicine.

No.	Activities or services
1	Taking best possible therapy history including both medications and CAM
2	Performing best possible patient therapy review including both medications and CAM
3	Performing therapy reconciliation at admission including both medications and CAM
4	Performing therapy reconciliation at transition of care including both medications and CAM
5	Performing therapy reconciliation at discharge including both medications and CAM
6	Identifying or resolving discrepancies or problems related to therapy including both medications and CAM
7	Providing collaborative, direct, or comprehensive patient care using medications and CAM
8	Developing therapeutic care plans including both medications and CAM
9	Participating in interprofessional discussions with regard to both medications and CAM
10	Making suggestions to other healthcare professionals with regard to both medications and CAM
11	Attending interprofessional meetings
12	Conducting patient education sessions with regard to both medications and CAM
13	Answering formal inquiries of other healthcare professionals concerning both medications and CAM
14	Reviewing therapy orders including both medications and CAM
15	Ordering, following up, or reviewing therapy monitoring orders including both medications and CAM
16	Identifying or resolving problems related to therapy contraindications with regard to both medications and CAM
17	Identifying or resolving problems related to therapy allergies with regard to both medications and CAM
18	Identifying or resolving problems related to therapy interactions with regard to both medications and CAM
19	Identifying or resolving problems related to food interactions with regard to both medications and CAM
20	Identifying or resolving problems related to inappropriate doses in patients with renal problems with regard to both medications and CAM
21	Identifying or resolving problems related to inappropriate doses in patients with hepatic problems with regard to both medications and CAM
22	Identifying or resolving problems related to therapy underdoses with regard to both medications and CAM
23	Identifying or resolving problems related to therapy overdoses with regard to both medications and CAM
24	Titrating doses of medications and CAM to produce desirable therapeutic effect
25	Identifying or resolving problems related to therapy adverse reactions with regard to both medications and CAM
26	Identifying or resolving problems related to therapy duplication with regard to both medications and CAM
27	Identifying or resolving problems related to ineffective therapy with regard to both medications and CAM
28	Identifying or resolving problems related to ambiguous orders including both medications and CAM
29	Identifying or resolving problems related to misspelled medications and CAM
30	Identifying or resolving problems related to illegibly written orders including both medications and CAM
31	Identifying or resolving problems related to missing orders including both medications and CAM
32	Identifying or resolving problems related to missing doses including both medications and CAM
33	Identifying or resolving problems related to missing frequencies of administration with regard to both medications and CAM
34	Identifying or resolving problems related to missing routes of administration with regard to both medications and CAM
35	Identifying or resolving problems related to missing duration of therapy with regard to both medications and CAM
36	Identifying or resolving problems related to missing recommendations to take medications and CAM in relation to meals
37	Identifying or resolving problems related to high alert medications or highly toxic CAM
38	Documenting assessments of response to therapeutic plan involving medications and CAM
39	Minimal number of complaints received
40	Minimal number of errors committed
41	Higher number of continuing education sessions attended
42	Higher number of continuing education sessions delivered

CAM: complementary and alternative medicine.

## Data Availability

Data supporting the results reported in this published article can be found in the Results section and Supplementary Materials.

## Supplementary Materials

Supplementary Materials Supplementary Table S1 shows adherence to the Preferred Reporting Items for Systematic Reviews and Meta-analyses statement for scoping reviews (PRISMA-ScR). Supplementary Table S2 shows the data extraction form. Supplementary Table S3 shows additional information on the studies included in this scoping review. Supplementary Figure S1 shows PRISMA flow diagram of study selection.
