# Supplementary material for: Quality Indicators of Pharmaceutical Care for Integrative Healthcare: A Scoping Review of Indicators Developed Using the Delphi Technique
Source: Evid Based Complement Alternat Med. 2020 Mar 19;2020:9131850. doi: 10.1155/2020/9131850 (PMC7106877; doi:10.1155/2020/9131850)
Supplement: Supplementary Materials — Supplementary Table S1 shows adherence to the Preferred Reporting Items for Systematic Reviews and Meta-analyses statement for scoping reviews (PRISMA-ScR). Supplementary Table S2 shows the data extraction form. Supplementary Table S3 shows additional information on the studies included in this scoping review. Supplementary Figure S1 shows PRISMA flow diagram of study selection. [file 9131850.f1.pdf]

Supplementary materials

Supplementary Table S1: Adherence to the Preferred Reporting Items for Systematic Reviews and Meta-Analyses statement for scoping reviews (PRISMA-ScR).

Supplementary Materials

Supplementary Table S1 shows adherence to the Preferred Reporting Items for Systematic Reviews and Meta-Analyses statement for scoping reviews (PRISMA-ScR) [1]

Section	Item #	PRISMA-ScR checklist item	Reported on page #
**Title**			
**Title**	1	Identify the report as a scoping review.	Identified as a scoping review. Please see the new title.
**Abstract**			
**Structured summary**	2	Provide a structured summary including, as applicable: background, objectives, eligibility criteria, sources of evidence, charting methods, results and conclusions that relate to the review question(s) and objective(s).	Provided and structured in accordance with the journal's style. Please see the abstract for background, objectives, inclusion criteria, data extraction, results, and conclusions.
**Introduction**			
**Rationale**	3	Describe the rationale for the review in the context of what is already known. Explain why the review question(s)/objective(s) lend themselves to a scoping review approach.	Provided. Please see the Introduction section, Page 4 Lines 1-8.
**Objectives**	4	Provide an explicit statement of the question(s) and objective(s) being addressed with reference to their key elements (e.g., population or participants, concepts and context), or other relevant key elements used to conceptualize the review question(s) and/or objective(s)).	Provided. Please see the Introduction section, Page 4 Lines 9-14.
**Methods**			

Section	Item #	PRISMA-ScR checklist item	Reported on page #
**Protocol and registration**	5	Indicate if a review protocol exists, if and where it can be accessed (e.g., web address), and, if available, provide registration information including registration number.	Protocol was not registered. Description of the protocol followed is provided with references. Please see Methods section, Page 5, Lines 5-6.
**Eligibility criteria**	6	Specify the characteristics of the sources of evidence (e.g., years considered, language, publication status) used as criteria for eligibility, and provide a rationale.	Provided. Please see the Methods section, Page 5, Lines 34-40.
**Information sources**	7	Describe all information sources (e.g., databases with dates of coverage, contact with authors to identify additional sources) in the search, as well as the date the most recent search was executed.	Provided. Please see the Methods section, Page 5, Lines 8-23.
**Search**	8	Present the full electronic search strategy for at least one database, including any limits used, such that it could be repeated.	Provided. Please see the Methods section, Page 5, Lines 8-23.
**Selection of sources of evidence**	9	State the process for selecting sources of evidence (i.e., screening, eligibility) included in the scoping review.	Provided. Please see the Methods section, Page 5, Lines 25-32.
**Data charting process**	10	Describe the methods of charting data from the included sources of evidence (e.g., piloted forms; forms that have been tested by the team before their use, whether data charting was done independently, in duplicate) and any processes for obtaining and confirming data from investigators.	Provided. Please see the Methods section, Page 6, Lines 9-21.
**Data items**	11	List and define all variables for which data were sought and any assumptions and simplifications made.	Provided. Please see the Methods section, Page 6, Lines 9-21.
**Critical appraisal of individual sources of evidence**	12	***If done,*** provide a rationale for conducting a critical appraisal of included sources of evidence; describe the methods used and how this information was used in any data synthesis (if appropriate).	Provided. Please see the Methods section, Page 6, Lines 9-21.
**Summary measures**	13	*Not applicable for scoping reviews.*	

Section	Item #	PRISMA-ScR checklist item	Reported on page #
**Synthesis of results**	14	Describe the methods of handling and summarizing the data that were charted.	Provided. Please see the Methods section, Page 5, Lines 25-32 and Page 6 Lines 9-21.
**Risk of bias across studies**	15	*Not applicable for scoping reviews.*	
**Additional analyses**	16	*Not applicable for scoping reviews.*	
**Results**			
**Selection of sources of evidence**	17	Give numbers of sources of evidence screened, assessed for eligibility, and included in the review, with reasons for exclusions at each stage, ideally using a flow diagram.	Provided. Please see the Results section, Page 7, Lines 3-7 and Supplementary Figure S1 for the PRISMA flow chart.
**Characteristics of sources of evidence**	18	For each source of evidence, present characteristics for which data were charted and provide the citations.	Provided. Please see the Results section, Page 10-15, Table 1 and Supplementary Table S3. Again, please see the narrative synthesis in the results section.
**Critical appraisal within sources of evidence**	19	***If done***, present data on critical appraisal of included sources of evidence (see item 12).	Please see Table 1 and the narrative synthesis in the results section.
**Results of individual sources of evidence**	20	For each included source of evidence, present the relevant data that were charted that relate to the review question(s) and objective(s).	Please see Table 1 and the narrative synthesis in the results section. Again, please see the Supplementary Table S3.
**Synthesis of results**	21	Summarize and/or present the charting results as they relate to the review question(s) and objective(s).	Provided. Please see the Results section, Table 1, Figures 1-4 and the supplementary Tables.
Risk of bias across studies	22	*Not applicable for scoping reviews.*	
Additional analyses	23	*Not applicable for scoping reviews.*	
**Discussion**			
Summary of evidence	24	Summarize the main results (including an overview of concepts, themes, and types of evidence available), explain how they relate to the review question(s) and objectives, and consider the relevance to key groups.	Provided. Please see the Discussion section, Page 25, Lines 2-9.
Limitations	25	Discuss the limitations of the scoping review process.	Provided. Please see the Discussion section, Page 26, Lines 21-37.
Conclusions	26	Provide a general interpretation of the results with respect to the review question(s) and objective(s), as well as potential implications and/or next steps.	Provided. Please see the Discussion section, Page 26, Lines 39-43.

Section	Item #	PRISMA-ScR checklist item	Reported on page #
**Funding**			
Funding	27	Describe sources of funding for the included sources of evidence, as well as sources of funding for the scoping review. Describe the role of the funders of the scoping review.	Provided at the end of the manuscript

Supplementary Table S2: Data extraction form

#	Author(s)	Publication year	Setting and/or country	Objectives of the study	Study design	Participants	Data collection	Analysis	Main results	Funding
1										
2										
3										
4										
5										
6										
7										
8										
9										
10										
11										
12										
13										
14										
15										
16										
17										
18										
19										
20										
21										
22										
23										
24										
25										

#	Author(s)	Publication year	Setting and/or country	Objectives of the study	Study design	Participants	Data collection	Analysis	Main results	Funding
26										
27										
28										
29										
30										
31										

Supplementary Table S3: Additional information on the studies included in this scoping review

#	Author(s)	Publication year	Setting and/or country	Study design	Analysis	Funding
**Pharmaceutical services relevant to medications**						
1	Fernandes et al	2015	Canada	A modified online Delphi technique of 3 rounds with an in-person meeting of the panelists that was held between the Delphi rounds 2 and 3	Qualitative comments and votes of the panelists on a Likert-scale of 1-9. `	Support from academic institutions and professional associations
2	Shawahna	2019	Palestine	A modified Delphi technique	Percentages, medians, and interquartile ranges of the votes of the panelists on a Likert-scale of 1-9. Votes of the panelists were compared using Dunn's multiple comparisons tests.	The study was funded by an academic institution
3	Krzyżaniak et al	2018	Poland	A modified Delphi technique of two consecutive online rounds	Votes of the panelists on a Likert-scale of 5 points. Percentages and frequencies of the votes of the panelists were analyzed.	Not funded
4	Cillis et al	2018	Belgium	A narrative review followed by 2 focus groups and a three-round Delphi technique	Votes of the panelists on a Likert-scale of 5 points. Mean vote of the panelists was used to define consensus.	Not funded
5	Ng and Harrison	2010	New Zealand	Surveys with a Delphi technique	Views of the participants on a Likert-scale of 1-5.	Not funded
6	Lima et al	2019	Brazil	A methodological study with quantitative approach using the Delphi technique	Votes on a Likert-scale of 5 points. Item content validity index and Fleiss kappa. The questionnaire was assessed for internal consistency reliability. Factor analysis with Oblimin rotation and Cronbach's alpha. Views of the pharmacists and experts were compared using the Mann-Whitney *U* test.	Funded by coordination for improving higher education personnel
7	De Bie et al	2011	The Netherlands	A literature review with a two round Delphi technique followed by a field test	Votes of the participants on a Likert-scale of 1-9.	Not declared
8	Grey et al	2016	United Kingdom	A postal survey followed by in-depth case studies and a two-round Delphi technique	The in-depth case studies were analyzed thematically. The panelists voted in the Delphi technique on a Likert-scale of 1-9.	The study was funded by a pharmaceutical trust for education
9	Clay et al	2019	United States	Literature review with a modified Delphi technique	Votes of the stakeholders.	Not declared
10	Richardson	2001	United Kingdom	A survey followed by a modified Delphi technique	Percentage of the panelists voting agreement.	Not declared
11	Mackinnon and Hepler	2002	Canada and United States	Literature review followed by a two round modified Delphi technique	Percentage of the panelists voting agreement.	Not funded
12	Pyne et al	2008	United States	Literature review followed by a modified Delphi technique	Votes of the panelists, medians, and ranges of votes.	Funded by government agency
13	Morris and Cantrill	2002	United Kingdom	A two round Delphi technique	Ratings of the panelists	Funded by an academic institution
14	Morris et al	2002	United Kingdom	Preliminary validation of the indicators followed by a two-round Delphi technique	Votes of the panelists on a Likert-scale of 1-7. Percentage of the panelists voting agreement.	Not declared
15	Robertson and MacKinnon	2002	Canada	A two round Delphi technique followed by a focus group	Votes of the panelists on a scale of 1-5.	Funded by a health institution

#	Author(s)	Publication year	Setting and/or country	Study design	Analysis	Funding
16	Currie et al	2003	United States	Literature review followed by a three round Delphi technique and group meetings	Votes of the panelists on a Likert-scale of 1-5.	Funded by a professional association
17	Malone et al	2004	United States	Literature review with a modified Delphi technique	Votes of the panelists on a Likert-scale of 1-10. Mean, standard deviation, and range of scores.	Government agency
18	Puumalainen et al	2005	Finland	Delphi technique	Votes of the panelists on a Likert-scale of 1-5. Percentage of the panelists voting agreement.	Not declared
19	Byrne et al	2010	Canada	A modified Delphi technique	Votes of the panelists on a Likert-scale of 1-5.	Health institutions
20	Bowie et al	2012	United Kingdom	Small groups, workshops, modified Delphi technique, and interviews.	Votes on a Likert-scale of 4 points.	Funded by a government agency
21	Fernandez-Llamazares et al	2013	Spain	A two round Delphi technique	Percentage of the panelists voting agreement.	Professional association
22	Floor-Schreudering et al	2014	The Netherlands	A two round Delphi technique	Votes of the panelists on a Likert-scale of 1-9.	Not declared
23	Tonna et al	2014	United Kingdom	A Delphi technique	Percentage of the panelists voting agreement.	Academic institution
24	Aljamal et al	2016	United Kingdom	A modified Delphi technique	Votes of the panelists on a Likert-scale of 1-9.	Not funded
25	Satibi et al	2019	Indonesia	Literature review followed by a modified three round Delphi technique	Votes of the panelists on a Likert-scale of 1-7.	Government institution
26	Rocha et al	2020	Brazil	A mixed method of iterations, meetings, and a Delphi technique	Votes of the panelists on a Likert-scale of 1-5.	Research organization
**Pharmaceutical services relevant to CAM**						
1	Im et al	2018	South Korea	A mixed method of qualitative and quantitative study design with preliminary studies	A Likert-scale of 1-5.	Academic institution
2	van Overveld et al	2017	The Netherlands	Literature review followed by a modified Delphi technique	A Likert-scale of 1-9.	Academic institution
3	Shawahna et al	2018	Palestine	Literature review and interviews followed by a two round Delphi technique	Votes of the panelists on a Likert-scale of 1-9.	Not funded
4	Shawahna and Al-Atrash	2019	Palestine	Interviews, literature review followed by a two round Delphi technique	Votes of the panelists on a Likert-scale of 1-9.	Not funded
5	Guangyi et al	2009	China	A three round Delphi technique	A Likert-scale of 1-5.	Academic institution

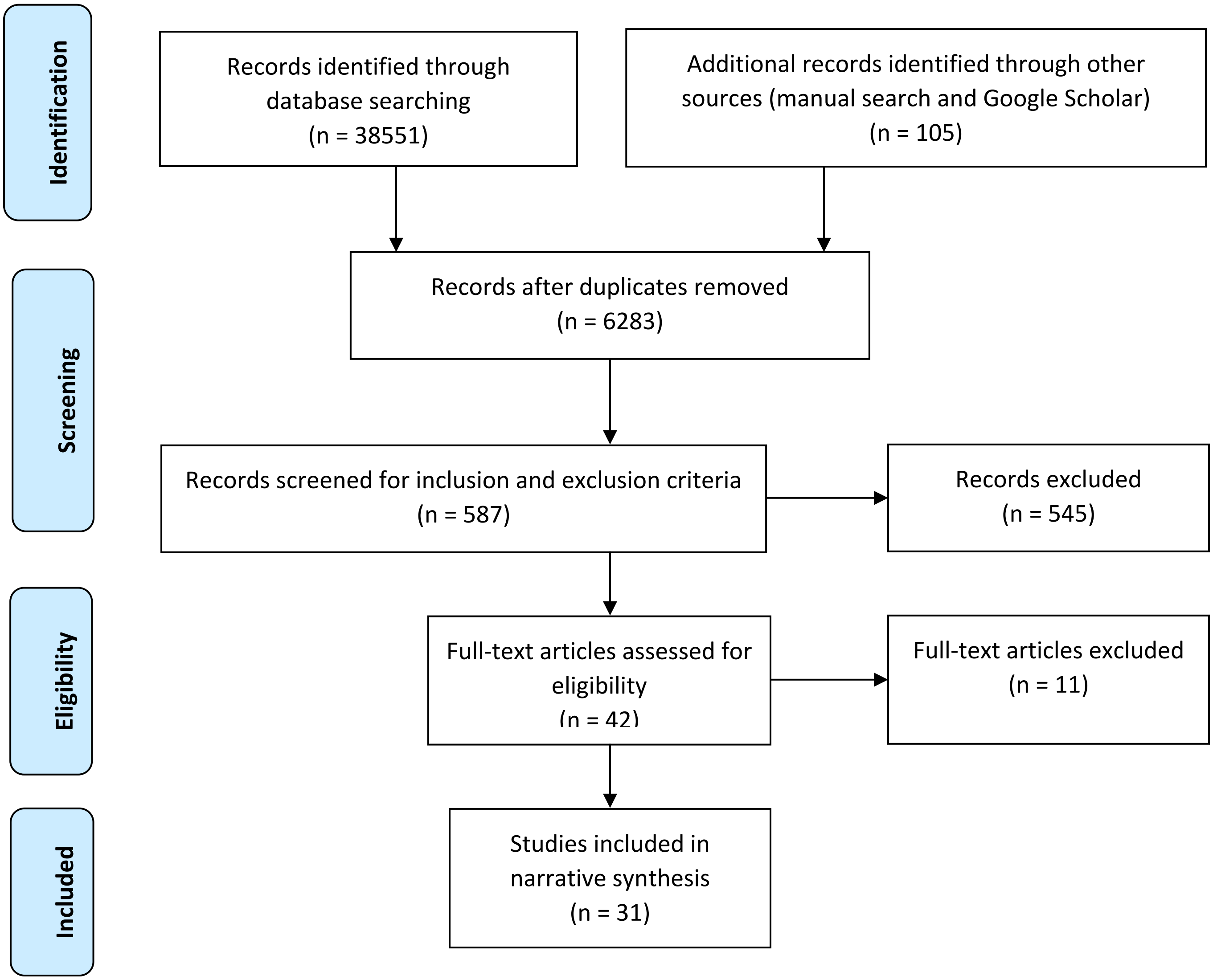

Supplementary Figure S1: PRISMA flow diagram of study selection

Reference

1. A. C. Tricco, E. Lillie, W. Zarin, et al., "PRISMA Extension for Scoping Reviews (PRISMA-ScR): Checklist and Explanation," *Ann Intern Med*, vol. 169, no. 7, pp. 467-473, 2018.
